# Biological network analysis with deep learning

**DOI:** 10.1093/bib/bbaa257

**Published:** 2020-11-10

**Authors:** Giulia Muzio, Leslie O’Bray, Karsten Borgwardt

**Affiliations:** Machine Learning and Computational Biology Lab at ETH Zürich; Machine Learning and Computational Biology Lab at ETH Zürich; Life Sciences at ETH Zürich

**Keywords:** deep learning, biological networks, protein function prediction, protein interaction prediction, drug development, drug-target prediction

## Abstract

Recent advancements in experimental high-throughput technologies have expanded the availability and quantity of molecular data in biology. Given the importance of interactions in biological processes, such as the interactions between proteins or the bonds within a chemical compound, this data is often represented in the form of a biological network. The rise of this data has created a need for new computational tools to analyze networks. One major trend in the field is to use deep learning for this goal and, more specifically, to use methods that work with networks, the so-called graph neural networks (GNNs). In this article, we describe biological networks and review the principles and underlying algorithms of GNNs. We then discuss domains in bioinformatics in which graph neural networks are frequently being applied at the moment, such as protein function prediction, protein–protein interaction prediction and *in silico* drug discovery and development. Finally, we highlight application areas such as gene regulatory networks and disease diagnosis where deep learning is emerging as a new tool to answer classic questions like gene interaction prediction and automatic disease prediction from data.

## Introduction

Understanding many biological processes requires knowledge not only about the biological entities themselves but also the relationships among them. For example, processes such as cell differentiation depend not only on which proteins are present, but also on which proteins bind together. A natural way to represent such processes is as a graph, also called a network, since a graph can model both entities as well as their interactions.

Recent advances in experimental high-throughput technology have vastly increased the data output from interaction screens at a lower cost and resulted in a large amount of such biological network data [1]. The availability of this data makes it possible to use biological network analysis to tackle many exciting challenges in bioinformatics, such as predicting the function of a new protein based on its structure or anticipating how a new drug will interact with biological pathways. This wealth of new data, combined with the recent advances in computing technology that has enabled the fast processing of such data [2, p. 440], has reignited interest in neural networks [3–6], which date back to the 1970s and 1980s, and set the stage for the emergence of deep neural networks, a.k.a deep learning, as a new way to address these unsolved problems.

Deep learning is a neural network comprised of multiple layers with (often non-linear) activation functions, whose composition is able to model non-linear dependencies. This has shown empirically strong performance in multiple fields, such as image analysis [7] and speech recognition [8]. One of the strengths of deep learning is its ability to detect complex patterns in the data, making it well suited for application in bioinformatics, where the data represent complex, interdependent relationships between biological entities and processes, which are often intrinsically noisy and occurring at multiple scales [9]. Furthermore, deep learning methods have been extended to graph-structured data, making it a promising technology to tackle these biological network analysis problems. The early examples of applying deep learning to biological network data, detailed in this paper, have consistently reported comparable or better results than the existing classical machine learning methods, highlighting its potential in the field.

We begin this paper by introducing biological networks and describing typical learning tasks on networks. Subsequently, we will explain the core concepts underpinning deep learning on graphs, namely graph neural networks (GNNs). Finally, we will discuss the most popular application tasks for GNNs in bioinformatics.

### Biological networks

DNA, RNA, proteins and metabolites have crucial roles in the molecular mechanisms of the cellular processes underlying life. Studying their structure and interactions is fundamental for a variety of reasons, including the development of new drugs and discovery of disease pathways. Both the structure and interactions of these entities can be represented using a graph, which is comprised of a set of nodes and a set of edges representing the connections between nodes. For example, molecules can be represented as a graph, where the nodes are the atoms and the edges are the bonds between the atoms. Similarly, many biological processes can be modeled with the entities as nodes and the interactions or relationships among them as edges. The aforementioned representation as a graph is convenient for a variety of reasons. Networks provide a simple and intuitive representation of heterogeneous and complex biological processes [10]. Moreover, it facilitates modeling and understanding complicated molecular mechanisms through the use of graph theory, machine learning and deep learning techniques.

As seen above, it is possible to define biological networks at different levels of detail. Besides the graph representation of biological actors used in investigating molecular properties and functions, other common biological networks include protein–protein interaction (PPI) networks, gene regulatory networks (GRN) and metabolic networks. Additionally, because of their relevance in contemporary health research, the above definition of a biological network is extended to include drug–drug interaction (DDI) networks. In the following, we provide a brief introduction to each of these networks.

**Protein-Protein Interaction Networks** PPI networks represent the interactions among proteins [11]. PPIs are essential for almost all cellular functions [12], ranging from the assembly of cell structural components, i.e. the cytoskeleton, to processes such as transcription, translation and active transport [13]. PPIs also include transient interactions, i.e. protein complexes that are formed and broken easily [14]. In PPI networks, nodes correspond to proteins while the edges define the interaction among connected proteins [15]. An exhaustive graph representation of PPIs would include also the type of the interaction, i.e. phosphorylation, or bond. However, in practice this is rarely captured.

**Gene Regulatory Networks** A GRN represents the complex mechanisms that regulate gene expression, the set of processes which leads to generating proteins from the DNA sequence [16]. Regulation mechanisms occur at different stages of protein production from DNA, such as during the transcription, translation and splicing phases. An intuitive explanation of these complex and interconnected mechanisms sees proteins both as the product and the controller of the gene expression [13]. In GRNs, each node represents a gene, and a directed link among two genes implies that one gene directly regulates the expression of the other without mediation from other genes [17].

**Metabolic Networks** Metabolic networks use graphs to represent metabolism, the set of all chemical reactions that occur within a living organism to maintain life. Metabolic actors are called metabolites, and they represent the intermediate and final products of metabolic reactions. Given their complexity, metabolic networks are usually decomposed into metabolic pathways, i.e. series of chemical reactions related to perform a specific metabolic function [18]. The graph representation of metabolism consists of mapping each metabolite to a node and each reaction to a directed edge labeled with the enzyme acting as the catalyst [19].

**Drug–Drug Interaction Networks** The objective of DDI networks is to model the interactions among different drugs [20]. A DDI network provides drugs as nodes and represents their interactions as edges. Unlike the previous networks, a DDI network does not represent a biological process. However, since it is a meaningful representation of knowledge about drug interactions, DDI networks are of increasing interest to researchers nowadays. Indeed, DDI networks are widely investigated for polypharmacy research [21].

As we have seen, biological networks are a rich way of representing biological data because they capture information not only about the entity itself but also the relationship between those entities. A large amount of information about these networks is already available, and we report on some of the most relevant biological network resources used in the reviewed methods in Table 1. Besides being an effective representation of a biological process, biological networks also unlock a suite of methods available for drawing new insights from graph data. We will introduce the classical types of problems that can be formulated on such graph-structured data in the following section.

**Table 1:** Resources of the most common biological networks that were used in the reviewed methods. We report the name, a short description, the website and which of the reviewed methods use them. The description indicates if the resource is a dataset (and therefore easy downloadable) or if it is a database accessible via web interface. The DrugBank database is included in two sections since it is used to collect the drug chemical structure and the information about DDIs.

	**Database**	**Description & website**		**References**
	Drug Repurposing Hub [22]	Curated database of FDA-approved drugs and clinical as well as pre-clinical chemical compounds		[23]
		*https://clue.io/repurposing*		
	DrugBank [24]	Database of drug structure, drug-target information and DDIs		[25–28]
		*https://www.drugbank.ca*		
	MUTAG [29]	Benchmark dataset reporting the molecular structure of 188 nitro compounds labeled as mutagenic & non-mutagenic on a bacterium		[30]
		*http://graphlearning.io/*		
*Chemical compounds*	National Cancer Institute 1/109 (NCI1, NCI109) [31]	Benchmark datasets reporting the chemical structure of compounds showing activity against some cancer cell lines		[30]
		*http://graphlearning.io/*		
	PubChem’s BioAssay database (PCBA) [32]	Benchmark dataset of small molecules reporting their high-throughput-measured biological activities		[33]
		*http://moleculenet.ai/*		
	Predictive Toxicology Challenge (PTC) [34]	Benchmark dataset reporting the structure of 344 compounds classified as carcinogenic and non-carcinogenic on rats		[30]
		*http://graphlearning.io/*		
	Quantum-Machine 9 (QM9) [35]	Dataset of small organic molecules with the structure & various properties		[36]
		*http://quantum-machine.org/datasets*		
	Tox21 [37]	Benchmark dataset of compounds & their toxicity on some biological targets		[33]
		*http://graphlearning.io/*		
	DrugBank [38], [39]	Database of drug structure, drug-target information and DDIs		[25], [40], [27], [41], [28]
		*https://www.drugbank.ca*		
*DDI Networks*	Twosides [42]	Comprehensive database of DDIs with respect to millions of adverse reactions		[43–46]
		*http://tatonettilab.org/offsides/*		
*Gene regulatory networks*	DREAM4 [47], [48]	Datasets of gene expression time series data & associated ground truth GRN structure from the DREAM4 100-gene *in silico* network inference challenge		[49]
		*http://gnw.sourceforge.net/dreamchallenge.html*		
*Metabolic networks*	BioModels [50]	Database of mathematical models of biological & biomedical systems, such as the Systems Biology Markup Language models of metabolic pathways		[51]
		*https://www.ebi.ac.uk/biomodels/*		
	Kyoto Encyclopedia of Genes and Genomes (KEGG) [53]	Biological pathways database for multiple model organisms		[52]
		*https://www.genome.jp/kegg/*		
	Biological General Repository for Interaction	Curated database of PPIs for multiple model organisms		[54–56], [28]
	Datasets (BioGRID) [57]	*https://thebiogrid.org*		
	Database of Interacting Proteins (DIP) [58]	Curated database of PPIs for multiple model organisms		[59], [60]
		*http://dip.doe-mbi.ucla.edu*		
	High-quality INTeractomes (HINT) [61]	Curated database of PPIs for multiple model organisms		[62]
		*http://hint.yulab.org/*		
*PPI networks*	Human Integrated PPI	Web tool to generate context-specific human PPI networks		[60]
	rEference (Hippie) [63]	*http://cbdm-01.zdv.uni-mainz.de/∼mschaefer/hippie*		
	Human Protein Reference Database (HPRD) [66], [67]	Database of human PPIs from high-throughput experiments		[59], [64], [40], [28], [65]
		*www.hprd.org*		
	Molecular INTeraction (MINT) [68]	Curated database of PPIs for multiple model organisms		[28]
		*https://mint.bio.uniroma2.it/*		
	Protein Interaction Network Analysis (PINA) [69]	Curated database of PPIs for multiple model organisms		[28]
		*https://omics.bjcancer.org/pina*		
	STRING [70]	Database of PPIs and tool for obtaining functional enriched PPI networks for multiple model organisms		[71], [55], [72], [41], [46]
		*https://string-db.org*		
	Dobson & Doig (D&D) [73]	Benchmark dataset of 1178 protein structures		[30]
		*https://graphlearning.io*		
*Proteins*	Protein Data Bank (PDB) [74]	Database of 3-dimensional structure of proteins for multiple model organisms		[75], [76], [26]
		*https://www.rcsb.org/*		

### Learning tasks on graphs

Learning tasks on graphs are at a high level categorized into node classification, link prediction, graph classification and graph embedding, though as we will discuss, approaches designed for one task can often be adapted to address multiple tasks. We will now explain each task in more detail.

**Node Classification** A typical task in biological network analysis is predicting the unknown function of a protein based on the functions of its neighbors in a PPI network. This problem, called node classification [77], is important when an input graph contains some nodes with labels, but many without, and the goal is to classify the remaining unlabeled nodes in the network. This is typically solved through some form of semi-supervised learning, where the algorithm uses the entire network as input during training with the goal of classifying all nodes. Although all nodes will be classified, the loss is calculated only on the nodes with a true label during training, thereby learning from the nodes with labels in order to classify the remaining unlabeled ones.

**Link Prediction** Current knowledge of interactions in biological networks is often incomplete, such as which genes regulate the expression of another in GRNs. Predicting these missing edges, i.e. link prediction [78], is a common task when working with such data, since it can be used to predict additional edges in a graph, or in the case of a weighted graph, the edge weight itself. This is also often framed as a semi-supervised learning problem, where the known links in a graph are used to predict where additional links may be present, similar to the node classification setup. Alternatively, link prediction can also be framed as a supervised learning problem, where after an embedding is learned for nodes, a secondary model is trained to predict whether there is a link between a given pair of nodes.

**Graph Classification or Regression** When the biological network data is comprised of multiple individual networks, such as a dataset of the 3D structure of molecules, the objective becomes predicting properties of each network, such as a molecule’s solubility or toxicity. This task, called graph classification [79], takes a dataset of graphs as its input, and then performs classification (or regression) for each individual graph. This is most commonly a supervised learning problem.

**Graph Embedding** Graph embedding [80–82] has the goal of finding a lower-dimensional, fixed-size vector representation of a graph, such as a PPI network, or an element within a network, such as a protein. This is typically achieved through unsupervised learning. Given the usefulness of representing nodes or graphs as a fixed-size vector, which enables a graph to use any off-the-shelf machine learning algorithm, learning a graph embedding is often used as a pre-processing step before using a standard machine learning algorithm for a particular task.

As described above, the graph representation of biological data enables the formulation of many classical learning tasks. While the high-throughput technology available today has resulted in a huge amount of such data, it has further underscored the need for novel computational methods to process and analyze it. These methods need to be both efficient, given the quantity of data, as well as high performing, in order to effectively replace previous methods. Deep learning can address both needs: it offers scalability for time-consuming tasks and has the potential for strong classification performance, as evidenced by strong performance gains in other fields. In the next section, we will discuss the principles and fundamental algorithms behind the deep learning approaches used on biological networks.

## Graph neural networks

Deep learning methods operate on vector data, and since graph data cannot directly be converted to a vector, special methods are needed to adapt deep learning methods to work with graphs.

GNNs are a class of such methods that adapt neural network methods to work in the graph domain [83]. While the field of GNNs encompasses many different sub-architectures, such as recurrent GNNs [84, 85], spatial-temporal GNNs [86, 87] and graph autoencoders [83], we focus here on the ones that are currently used in biological network analysis, namely graph embedding techniques [80–82] and graph convolutional networks (GCNs) [83]. We note that although closely related to GNNs, graph embedding techniques are not always considered a subset of GNNs. However, network embedding is closely related and it is used frequently as one of the building blocks for the deep learning applications mentioned in this paper, so we will describe it under the umbrella categorization of GNNs. In this section, we will first present the critical notation used when working with graphs and present the fundamental graph embedding and GCN algorithms used in bioinformatics.

### Notation

We will refer to a graph $\mathcal{G} = (V, E)$, as the set of vertices $V$, with $|V|=n$, and the set of edges $E$, where $e_{ij} \in E$ indicates an edge between $v_i$ and $v_j$. Each graph $\mathcal{G}$ can be represented by its adjacency matrix $\textbf{A} \in \mathbb{R}^{n \times n}$. If the graph is unweighted and undirected, any edge $e_{ij}$ will be denoted by a $1$ at $\textbf{A}_{ij}$ and $\textbf{A}_{ji}$. Graphs with node attributes store these values in an additional matrix $\textbf{X} \in \mathbb{R}^{n \times d}$, where $d$ is the dimension of the node attributes. While this section deals primarily with homogeneous, unweighted and undirected graphs, it is worth noting the diversity of graph representations. Graphs can be heterogeneous, meaning that their nodes or edges can have multiple types, such as in a knowledge graph [88]. If $\mathcal{G}$ is a weighted graph, the entry for edge $e_{ij}$ in $\textbf{A}$ will be the edge weight $w_{ij}$, and if $\mathcal{G}$ is a directed graph, an edge $e_{ij}$ does not imply an edge $e_{ji}$, meaning $\textbf{A}$ is not necessarily symmetric.

**Figure 1:**
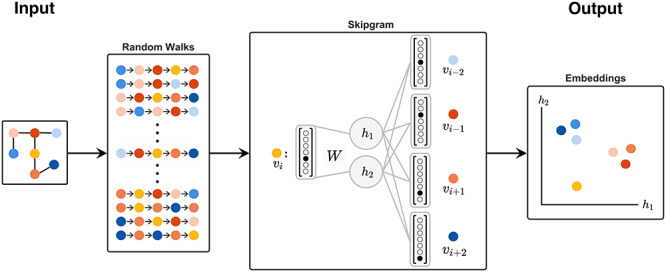
This shows the process of learning a simple graph embedding using DeepWalk. From an input graph, a fixed number of random walks are generated from each node with a predetermined length. The embeddings for each node are then learned using the Skipgram objective, where a node on the random walk is given as input to a single layer neural network. The input is compressed down to an $d$-dimensional representation (here, $d=2$) with an embedding matrix $W \in \mathbb{R}^{\mid V \mid \times d}$, and then used to predict which nodes surround it on the walk. That is, a node $v_i$ is used to predict the surrounding nodes on the walk within a given context window (here, size two): $v_{i-2}, v_{i-1}, v_{i+1}$ and $v_{i+2}$. After training, this lower dimensional representation for each node, which can be easily retrieved from $W$, is then used as the embedding for each node. Note that DeepWalk chooses the next node in the random walk uniformly at random, and therefore can return to previous nodes in the walk, whereas node2vec introduces a parameter to control the probability of doing so.

### Fundamental algorithms for deep learning on graphs

We will now detail two sub-fields that are widely used in bioinformatics today: graph embedding and GCNs, which in addition to being the most widely used architectures in bioinformatics, are the fundamental building blocks of many other GNN architectures. The algorithms that we will present can be used to solve the learning tasks presented in the introduction, namely node classification, link prediction, graph classification/regression and graph embedding.

#### Graph embedding

While graph embedding is often not strictly considered as a subset of GNNs, it is intertwined with them, and given its importance for other GNNs and bioinformatics, is considered in detail here. Graph embedding approaches seek to learn a low-dimensional vector representation of a graph or elements of a graph, such as its nodes. This embedding is typically then re-purposed for use in node or graph classification, or link prediction tasks.

While there are many approaches addressing the graph embedding problem, the most iconic are DeepWalk [89], node2vec [54] and LINE [90]. DeepWalk [89] utilizes the word2vec [91] framework from natural language processing to learn embeddings for each node in the graph by generating multiple random walks from each node and then optimizing a Skipgram objective function. The Skipgram training objective learns an embedding for a node such that it maximizes the probability of predicting the nodes that surround it in the random walk, in the same way that word2vec learns a word embedding that can predict the surrounding context words. More concretely, this can equivalently be formalized as the following minimization problem in Eq. 2 of [89]:

(1) \begin{align*} & \underset{\Phi}{min} \;\; -\log P({v_{i-w}, \ldots, v_{i-1}, v_{i+1}, \ldots, v_{i+w} | \Phi(v_i))}, \end{align*}

where $\Phi : v \in V \mapsto \mathbb{R}^{|V| \times d}$ maps each vertex into a $d$-dimensional space, resulting in a matrix of size $|V| \times d$, and $w$ is the size of the context window surrounding a node $v_i$. node2vec [54] expands upon the framework introduced by DeepWalk by introducing parameters to control whether the random walks are biased towards a depth-first search or a breadth-first search. LINE [90] takes a different approach. It seeks to learn a low-dimensional embedding such that the first- and second-order proximity of nodes, representing whether nodes are directly connected and whether they share common neighbors, respectively, are preserved. That is to say, nodes which are connected by an edge, or have similar sets of neighbors, should be close to one another in the embedded space. LINE is trained by minimizing an objective function that captures the first- and second-order proximity by asynchronous stochastic gradient descent. Once an embedding for the nodes or graph has been learned, pairs of nodes can be used as input in order to predict whether there is a link between them, as is done for example in node2vec.

#### Graph convolutional networks

GCNs are a subset of GNNs that adapt the highly successful convolutional neural network (CNN) architecture [92] to work on graph-structured data. Whereas CNNs, which are often used with images, are able to leverage the spatial information and relationships captured in an image, due to the fact that a set of images can be defined on the same regular grid, the ordering of a graph’s adjacency matrix is arbitrary, and thus cannot not directly translate to the CNN framework. GCN methods define and use a spectral- or spatial-based convolution over the graph, providing a graph domain analog to the image convolution in CNNs.

Spectral methods, first introduced by Bruna et al. [93] and later Defferrard et al. [94], build a convolution by creating a spectral filter defined in the Fourier domain using the graph Laplacian. However, due to the computational complexity of the eigendecomposition of the graph Laplacian necessary for spectral methods, many more methods have been developed using spatial methods, where the idea is to learn an embedding for each node by aggregating its neighborhood in each successive layer in the network. By using a permutation-invariant function for the aggregation step, such as the sum or the mean, one can circumvent the problem of the arbitrary ordering of an adjacency matrix, which was what prevents a graph from using a standard CNN. Each additional layer incorporates information from further out neighborhoods; the $k^{\textrm{th}} $ layer in the network corresponds to incorporating the $k$-hop neighborhood of a given node. Duvenaud et al. [95] was an early example of this, providing a permutation-invariant convolution that operates over all nodes in the graph, and in doing so, calculated the sum of the features of a node and its neighbors. While initially designed to retrieve a fixed size vector representation of a graph, i.e. an embedding of the graph, the actual method was trained on graph regression tasks.

Kipf and Welling [96] provide another spatial-based method, and is perhaps the most seminal example of GCNs, often considered to be the baseline example of GCNs. Its significance is also due in part to the fact that they bridge the gap between spatial and spectral methods by showing a spectral motivation for their spatial approach. Though this approach was originally proposed as a way to perform node classification via semi-supervised learning, it can be easily generalized to classify higher-order structures in the graph, edge level outcomes, or the graph itself. They define a propagation layer for the network, where each layer effectively incorporates information from that node’s $k$-hop neighborhood, as well as node features. This forward propagation of a two-layer network then takes the following form, generalized from Eq. 9 in [96]:

(2) \begin{align*} & Z = f(\textbf{X}, \textbf{A}) = \textrm{softmax} (\hat{\textbf{A}} \sigma \big( \hat{\textbf{A}}\textbf{XW}^{(\textbf 0)} )\textbf{W}^{\textbf{(1)}}), \end{align*}

where $\hat{\textbf{A}} \in \mathbb{R}^{n \times n}$ is the normalized adjacency matrix with added self-loops, derived from original adjacency matrix $\textbf{A}$, $\textbf{X} \in \mathbb{R}^{n \times d}$ is the feature matrix containing node attributes of all $n$ nodes, $\textbf{W}^{(\textbf{i})}$ are the weights from the $i^{\textrm{th}}$ layer and $\sigma $ is an element-wise activation function, such as $ReLU = \max (\cdot , 0)$. The output of the model, $Z$, in this example represents the class probabilities for each node, therefore $Z \in \mathbb{R}^{n \times c}$, where $c$ is the number of classes. If $h$ is the number of hidden units, then $\textbf{W}^{(\textbf{0})} \in \mathbb{R}^{d \times h}$ and $\textbf{W}^{(\textbf{1})} \in \mathbb{R}^{h \times c}$.

Hamilton et al. [64] posit a similar idea with their GraphSAGE algorithm, but with the goal of learning a more generalizable and computationally efficient approach to the problem. While the initial goal is node embedding, this is again done with the end goal of another task, such as node classification or link prediction. They achieve this speedup by sampling a node’s neighbors, rather than taking the entire neighborhood, and by learning an aggregation function, for which they considered the mean, max and long-short term memory aggregator functions.

**Figure 2:**
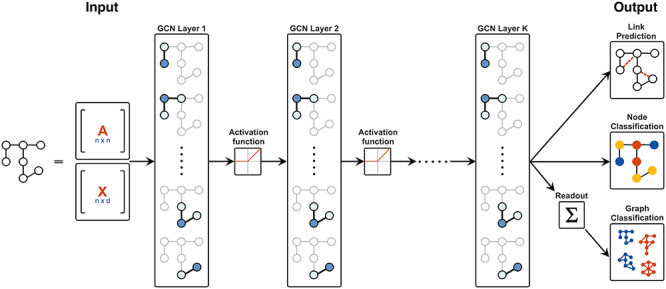
A visual depiction of a $k$-layer GCN. The input is the adjacency matrix $\textbf{A} \in \mathbb{R}^{n \times n}$ of a graph and node attribute matrix $\textbf{X} \in \mathbb{R}^{n \times d}$. Each layer of the GCN aggregates over the neighborhood of each node, using the node representations from the previous layer in the network. The aggregations in each layer then pass through an activation function (here, $ReLU$) before going to the next layer. This network can be used to produce various different outputs: for predicting new edges in the input network (link prediction), classifying individual nodes in the input graph (node classification), or classifying the entire input graph (graph classification). In order to perform graph classification, an additional readout step (here, the sum over all nodes) is required to map the output from $\mathbb{R}^{n \times c}$ to $\mathbb{R}^{c}$. The color represents the predicted classes for the respective entity in the output.

Gilmer et al. [36] provide an interpretation of graph convolutions from a message passing point of view, where each node sends and receives messages from its neighbors, and in doing so is able to update the node state. At the end of the network there is a readout step that aggregates the node states to the appropriate level of output (e.g. from the node level to the graph level). Impressively, Gilmer et al. are able to show the direct translation of many of the papers mentioned here into their framework, and thus their neural message passing has become a leading paradigm in GNNs today. Furthermore, they test out various configurations of such a scheme and show the best configuration to predict molecular properties.

These approaches to GCNs can also be understood as a neural network analog to the Weisfeiler–Lehman kernel for measuring graph similarity [97, 98], which is based on the classic Weisfeiler–Lehman test of isomorphism [99], a comparison which Kipf and Welling [96] and Hamilton et al. [64] make explicitly. By aggregating over all neighbors of a node, using the identity matrix for $\textbf{W}$, and setting $\sigma $ to an appropriate hash function, one effectively recovers the Weisfeiler–Lehman algorithm. The adaptations in GCNs can therefore be seen as a differentiable and continuous extension of the Weisfeiler–Lehman algorithm and kernel.

In an entirely different approach to deep learning on graphs, Niepert et al. [30] solve the node correspondence problem by imposing an ordering upon the graph, and in doing so opens the door to utilize a more traditional CNN structure. Rather than using the full graph as input, it defines a common fixed-size representation for all graphs. The entries in the grid are filled by the $j$ most important nodes in a graph, according to some predefined importance measure, as well as the $k$ closest neighbors of each of the $j$ nodes. Any corresponding node and/or edge attributes associated with the nodes in question can also be included. In doing so, graphs of different sizes are all standardized to the same size grid, which enables learning using a standard CNN filter.

In all these approaches, training is done by iteratively calculating a task-specific loss function over all relevant samples (such as the nodes with labels or the graphs). The loss is then propagated back through the network via backpropagation. The gradient of the weights $\textbf{W}$ are calculated and $\textbf{W}$ is correspondingly adjusted according to a pre-defined update equation.

## Applications in biology

In reviewing the different applications of deep learning on biological networks, we encountered varying degrees to which network information was included. We therefore had to define what constituted deep learning on a biological network. From the deep learning point of view, we defined this as learning approaches based on a hierarchy of non-linear functions. This review accordingly focuses on deep learning methods and does not summarize methods using classic machine learning algorithms, such as kernel methods, SVMs, random forests, etc, though we will discuss how the new deep learning methods perform relative to the classic counterparts. Secondly, we had to define what qualified as a biological network, since some methods can use features of a graph without explicitly leveraging the graph structure. As an example, one could build a feature vector based on the node label counts of amino acids in a protein. Whether to include an example such as this is not always straightforward. We ultimately decided to include any method that explicitly discussed or generated features from the graph properties as valid methods.

We will now discuss some of the main use cases of biological network analysis and deep learning. We begin with the more established practices, namely in protein analysis and drug development and discovery. We will then discuss the application areas in which deep learning is emerging as a competitive alternative to current methods, such as in disease diagnosis and the analysis of gene regulatory and metabolic networks. We provide information about the implementations of the various methods in Table 2 in the Supplementary Materials. In general, the performance of the reviewed methods have been assessed using a classic cross validation framework. Some papers go even further and use an additional external validation dataset to test the generalizability of the proposed approach. Furthermore, some works even validate the *de novo* prediction through literature research or by performing lab experiments. When either of these is the case, it is explicitly mentioned.

### Proteomics

Proteins play a pivotal role in many biological processes, and thus better understanding their roles and interactions with one another is critical to answering a variety of biological questions. Deep learning has emerged as a promising new way to answer some of these classic questions. In this section we will focus on three main categories of deep learning tasks on proteins: predicting whether a pair of proteins will interact, determining the function of a given protein and predicting the 3D structure of proteins.

#### Protein interaction prediction

As mentioned in the introduction, nodes in a PPI network are proteins and the edges between nodes represent an interaction. Given a graph of proteins with edges representing known protein interactions, the goal is to predict what other pairs of proteins in the graph are also likely to interact. From a graph-theoretic point of view, this is a link prediction problem. Using GCNs enables these methods to directly incorporate network information, which is typically not included in classical machine learning methods. Traditionally, many methods use the primary structure of amino acid sequences in order to vectorize a protein and perform classification. However, the recent methods that leverage the graph structure have shown stronger performance compared to merely using the sequence information and is discussed in more detail below.

As a broader assessment of classic approaches, Yue et al. [41] evaluate state-of-the-art network-based methods from other fields on bioinformatics tasks, to provide a baseline performance from which the field should be improving upon. The approaches generally combine a network embedding with another deep learning approach in order to assess its performance on predicting links in a PPI network and concluded that the more recent neural network based embedding approaches showed the most potential on bioinformatics tasks, and outperformed the traditional methods.

Liu et al. [60] augment protein interaction prediction from a pure sequence-based vector approach to one that also incorporates network information using a GCN. They propose learning a representation of each node by using a generic GCN framework on a PPI with an encoding of the primary structure sequences of the protein. The representations of each pair of proteins are later used as the input to a deep neural network to predict whether a pair of proteins will interact. This approach extends the previous work of DeepPPI [59], which used deep learning on a vector summary of the protein sequences to predict links. DeepPPI outperformed classical methods such as SVM, random forest, and naive Bayes, across a variety of metrics including accuracy, precision and recall. Liu et al.’s model surpassed even DeepPPI’s performance, showing the value of incorporating the network information into the model.

Zhang and Kabuka [100] attempt to capture the complexity of protein data and directly use topological features by incorporating multiple modalities of the data, such as the first and second order similarity, and the homology features extracted from protein sequences. They pre-process the data by forming a vector summary for each protein based on features such as the amino acid composition and then use a combination of unsupervised and supervised learning approaches to predict the interaction. Besides having better accuracy and precision compared to classical methods such as nearest neighbor and naive Bayes, they also showed that their state-of-the-art prediction performance method was maintained across datasets from eight different species.

#### Protein function prediction

Another area of protein analysis lies in predicting the function of a protein, given that manual assessment of the large amounts of data resulting from high-throughput experiments is rather slow and costly. There are two typical ways in which this question is posed: as a node classification task or a graph classification task. As we will discuss below, the new deep learning methods reviewed here are typically compared to the state-of-the-art methods based on classical machine learning approaches and report to outperform them.

**Node Classification** In a node classification approach, the input is a PPI where only the function of some nodes (i.e. proteins) is known. The task is to classify the unknown nodes’ function. Some of the previously discussed methods in predicting PPIs were also used to classify the nodes in the network. For example, two of the classic GCN algorithms described in in the Section “Graph neural networks,” GraphSAGE [64] and node2vec [54], were validated on PPI datasets and used to predict the function of proteins within the network. Additionally, Zhang and Kabuka’s approach [100] to predict PPIs was also extended to classify the function of a given protein. Similarly, Yue et al. [41] also evaluate the performance of various network algorithms on the task of node prediction to predict the function of proteins.

In a new approach, Gligorijević et al. [71] consider the idea of representing PPI networks using multiple representations of the same network. Each network contains different information, but uses the same set of nodes. They create a vector representation of each node using Random Walk with Restarts from Cao et al. [101] to then construct a positive point-wise mutual information matrix for each of the adjacency matrices, which is used as the input to a multimodal deep autoencoder. The setup allows giving multiple PPIs as input and facilitates the integration of all this information, ultimately yielding a low-dimensional vector which is then given to a SVM for protein function classification. The authors found that using a deep learning autoencoder learned a richer and more complex vector embedding of a network, leading to better performance compared to the previous state-of-the-art methods based on classical machine learning methods.

Zeng et al. [56] seek to identify essential proteins from a PPI network. They learn a dense vector representation of each node using node2vec [54], and combine that with a representation learned from gene expression profiles using a RNN. This is then passed through a regular fully connected network, in order to classify each node as an essential or non-essential protein. They again compare their methods with classic machine learning approaches such as an SVM, decision trees, and random forests, and find their method outperforms all of them across metrics such as accuracy, recall and AUC. Furthermore, through an ablation study the authors revealed the most critical component of their method driving the performance was the network embedding of the PPI, showcasing the valuable information that is captured in a network.

OhmNet [65] provides yet another approach, which learns representations of nodes in an unsupervised manner by using multiple layers of PPI networks generated from different tissues. This provides a more informative view into cellular function by incorporating the differences across tissues. The representation is learned based on the network architecture, in an extension to node2vec [54] for multi-scale graphs, which is later used to classify the protein function in the network. They compare themselves against classic methods such as methods based on tensor factorization and SVMs, as well as to some of the baseline network embedding methods like LINE and node2vec, and found superior performance to all of them in terms of AUROC and AUPRC. They attribute the benefit from having their multi-scale view of the proteins across tissues, which previous methods often modeled as a single network.

**Graph Classification** The second type of approach takes the graph of a protein’s secondary structure elements as input and classifies it into a functional group. While there are many classical methods that tackle this problem, as in [102], deep learning offers an alternative way to address the problem. Several of the classic GCN methods mentioned in the Section “Graph neural networks” use protein function prediction as an application of their method, such as Niepert et al. [30]. The formulation of the question is quite similar to that of drug properties prediction, discussed further in the subsection “Prediction of drug properties” except that the task is classification rather than regression. Given the strong overlap, we leave the discussion of specific methods to that subsection.

#### Protein structure prediction

A related problem to protein function prediction is protein structure prediction. Since the 3D structure of a protein largely informs its function, these two problems are interlinked. Recent work has focused on developing methods to predict the 3D structure of a protein from its genetic sequence, also known as the protein folding problem. Although there were previous efforts to use deep learning to predict residue contact to help solve the protein folding problem [103, 104], AlphaFold [76] represents a groundbreaking approach that set a new baseline substantially above both deep learning and traditional approaches and is thus the only article we discuss in detail here. AlphaFold, like other approaches, begins with the sequence of amino acids as the basis upon which it will predict the 3D structure. This input is combined with other feature information gathered from protein databases, and uses a CNN to predict the discrete probability distribution of the distances between all pairs of amino acids, as well as the probability distribution of the torsion angles. Predicting the distance and its corresponding distribution yielded more informative and accurate results compared to previous approaches which just predict whether two residues were connected by a link. The authors used the distances and the torsion angles, in conjunction with a penalty if the prediction caused atoms to overlap, to assess the quality of their prediction, called the potential. They were then able to perform stochastic gradient descent to iteratively improve their model. Using this approach yielded unprecedented results, and gave insight into the potential that deep learning can have in addressing some of the most challenging bioinformatics problems.

### Drug development, discovery and polypharmacy

Deep learning has recently been used to improve two steps of the process of drug discovery and development [105], namely: (i) screening thousands of chemical compounds to find the ones that react with a previously identified therapeutic target, and (ii) studying the properties of the potential drug candidates, e.g. toxicity, or absorption, distribution, metabolism, and excretion (ADME). There is interest in improving the screening step since it is quite laborious, expensive and time-consuming. We begin this section reviewing papers that present deep learning methods as an alternative to the current manual screening process, often called drug-target prediction. Then, we summarize deep learning approaches whose aim is to predict drug properties. Subsequently, we discuss the increased interest focused on the identification of which combination of drugs, known as polypharmacy, can be effective for treating human diseases whose mechanisms are too complicated to be treated by using a single one [106]. However, this therapeutic can have undesired side effects due to the interaction among combination of drugs [107]. It is therefore crucial to identify DDIs, which is nearly impossible to do manually. We present the papers which try to address this problem by combining deep learning approaches with DDI networks.

#### Drug–target prediction

After the identification of a therapeutically relevant target, i.e. a protein, it is essential to properly determine its interactions with different chemical compounds to characterize their binding affinity, or drug-target interactions (DTIs). This testing process is usually referred to as a screening, and its output consists of a list of potential drug candidates showing high binding affinities with the target. As already mentioned, manual screening is expensive and time-consuming since it must be performed on thousands of molecules to find a single drug. Deep learning methods try to overcome this limitation, often using DDI networks. Drug-target interaction prediction within the graph deep learning framework is therefore typically formulated as a link prediction problem. Graph-based deep learning methods have shown that they are capable of effectively tackling the drug-target prediction problem across various methods, achieving superior performance to previous state-of-the-art methods.

Some of these methods follow a systemic approach, where several biological networks (PPIs, DDIs) are taken into account in order to solve the prediction problem. An interesting paper belonging to this category is from Manoochehri et al. [40], which proposes an encoder-decoder GCN to predict the interactions among potential drugs and a therapeutic target. The method takes an heterogeneous network composed of drugs, proteins, diseases, and side effects as input, where nodes can be drugs, proteins, or diseases. Edges exist when nodes are connected by a relationship whose interaction type determines the edge label, such as drug–drug and protein–protein similarities, drug–protein, drug–drug, protein–protein, drug–disease and drug–protein side effects interactions. The authors combine different data resources in order to construct this network. The encoder takes the described network as input, and returns an embedding of the nodes, which is used by the decoder to capture drug-protein interactions. The output of this procedure is the estimated likelihood of the existence of an edge between pairs of proteins and drugs. The stability and flexibility of the proposed method is evaluated on substantial variations of the heterogeneous network.

Zeng et al. [28] follow a similar systemic approach to solve the DTI prediction problem, proposing a method called deepDTnet. Both [40] and deepDTnet [28] outperform the state-of-the-art methods in the field. In addition, deepDTnet is compared to classic machine learning approaches, namely random forests, SVMs, k-nearest neighbors, and naive Bayes, and outperform them on an additional external validation set, demonstrating the generalizability of their method. Additionally, deepDTnet shows higher robustness in comparison to the baselines, since it performs well on drugs or targets showing high and low connectivity as well as high or low chemical similarity. deepDTnet’s predictions were further validated in a *in vivo* lab experiment.

Another category of methods characterizes the DTI by considering and analyzing the molecular structure of drugs and targets. In [26], the authors propose a GCN approach to the DTI prediction problem whose input consists of two graphs, a protein pocket graph and a 2D drug molecular graph. Their method is composed of two steps, namely (i) a preliminary unsupervised phase consisting of an autoencoder used for learning general pocket features, and (ii) a supervised graph convolutional binding classifier. The latter is composed of two GCN models working in parallel, i.e. a pocket and a drug GCN, which extract features from the protein pocket graph and the 2D molecule graph respectively. There is a layer responsible for the integration of interactions between proteins, generating a joint drug-target fingerprint, which are then classified into “binding” and “non-binding” classes. The authors compare their model with existing deep learning methods and docking programs popular in the field and report better performance. Results obtained on an external validation dataset showed the higher generalizability of [26] in comparison to the baselines.

Fout et al. [75] introduce another method to predict whether a given pair of proteins will interact for the purpose of drug-target prediction. In this approach, two graphs are given as input: the ligand protein and the receptor protein. The nodes in both graphs correspond to residues, and each node is connected to the $k$ closest other nodes determined by the mean distance between their atoms. Rather than simply predict whether a pair of proteins interact, this predicts where specifically on the protein it will interact. Their method is an extension of the fingerprint method introduced by Duvenaud et al. [95], but allows for different weighting of the center node vs. its neighbors by training different weights and enables the inclusion of edge features. This approach outperformed the other state-of-the-art method that was based on using an SVM.

Lastly, PotentialNet is a family of GCNs proposed by Feinberg et al. [108] which differs from the previous ones since it considers the non-covalent interactions among different molecules as input, in addition to the graph molecular structure. More specifically, the method includes three stages: (i) a graph convolution over covalent bonds only, (ii) a simultaneous covalent and noncovalent propagation which takes into account the spatial information between atoms and (iii) a graph gather step performed only on the ligand atoms, whose representation is derived from both bonded ligand information and spatial proximity to protein atoms. The cross validation strategy in [108] is particularly interesting, since it tests PotentialNet’s generalization capabilities by mimicking real DTIs prediction scenarios, e.g. predicting affinity properties on unseen molecules. Furthermore, PotentialNet is comparable to the classic machine learning state-of-the-art methods in molecular affinity prediction field.

**End-to-End Drug Discovery & Development** While the approaches described above solve just the screening step, Stokes et al. [23] recently introduced an approach to tackle the entire drug discovery and target validation step. Motivated by both the marked increase in antibiotic-resistant bacteria and the difficulty of discovering new antibiotics, the authors propose a deep learning approach to identify molecules showing growth inhibition against a target bacterium, namely *E. coli*. Their research is directed towards the discovery of candidates whose molecular structure is different from currently available and known antibiotics. Unlike the other drug-target prediction methods, this is a graph classification problem. A directed message passing neural network named Chemprop [109] is trained with a feature-enriched graph representation of molecules labeled according to their action against *E. coli*. Since the previous step mainly captures local properties, a global feature molecular representation [110] is also given to the classifier. After the learning step, the obtained classifier is deployed on several chemical libraries, containing more than 107 million molecules, to obtain a list of potential candidate compounds that could be antibacterial against *E. coli*. Then, the identified molecules are filtered according to the clinical phase of the investigation and to pre-defined scores penalizing similarity with training molecules and toxicity. This procedure led to the identification of halicin from the Drug Repurposing Hub. Halicin properties and action mechanisms were experimentally investigated and the results proved its antibacterial activity on *E. coli* and on other bacteria in mice, showing that deep learning can effectively improve the antibiotic discovery screening process in a more time and cost effective way.

#### Prediction of drug properties

After the screening step, which provides a list of molecules showing high affinity with the therapeutic target, the properties of these candidates have to be investigated. This becomes a graph classification or regression problem. We will review methods that seek to predict those properties, such as the absorption, distribution, metabolism and excretion (ADME), stability, solubility, toxicity and quantum properties of chemical compounds represented as graphs. The following methods are compared to the classic machine learning counterparts, with competitive results and are detailed below. This fact highlights the effectiveness of deep learning to capture meaningful information from the graph structure, and therefore its potential to provide an alternative to classic state-of-the-art methods for predicting drug properties.

ADME prediction is the objective of Chemi-Net [111], a method which combines a GCN with a multi-task deep neural network, which can simultaneously solve multiple learning tasks. Chemi-Net’s input is a molecule represented by two feature sets, describing atoms and atom pairs respectively. The first operation consists of the projection of the assembling of the atoms and atom pair descriptor onto a 3D space, to obtain a molecule-shaped graph structure. The latter undergoes a series of graph convolution operations whose output is then reduced to a single fixed sized molecule embedding during the readout step. ADME prediction is obtained after this last embedding representation passes through several fully connected layers. The authors compare the results obtained by employing the GCN’s embeddings, e.g. single-task learning Chemi-Net, with the ones achieved when using the traditional property descriptors. Chemi-Net outperforms the baseline on almost all datasets, except the small noisy ones. The authors overcome this limitation by means of a multi-task learning framework, which allows them to leverage the information enclosed in large datasets to compensate for the small ones.

Stability is another crucial property to be investigated in the drug discovery and development process. The method proposed in DeepChemStable [112] aims to predict the stability of chemical compounds from their graph representation by combining a GCN and an attention mechanism. The GCN is able to capture the molecular structure at a local level, while the attention mechanism learns the global graph information. DeepChemStable investigates which features cause the instability of the chemical compound, which enables it to obtain more interpretable results. The authors contrast DeepChemStable with a naive Bayes-based baseline, showing the potential of the proposed deep learning framework. DeepChemStable and the baseline are comparable in terms of AUC and precision, while DeepChemStable is superior in terms of recall. PotentialNet [108], introduced in the subsection “Drug-target prediction” for DTI prediction, has further applications in drug molecular properties prediction, where its performance is also competitive or superior to existing methods.

Additionally, several of the fundamental GCN algorithms tried to address the problem of drug property prediction. As discussed earlier, Duvenaud et al. [95] propose a neural network based approach for finding a fingerprint for each molecule, which is then used to predict drug properties of molecules such as solubility, drug efficacy and organic photovoltaic efficiency of molecules and showed improved performance relative to the state-of-the-art circular fingerprint method. Kearnes et al. [33] expand upon this idea by performing convolutions on edge information in addition to the node information. The Patchy-San algorithm by Niepert et al. [30], also previously discussed, was also used to classify molecules according to their carcinogenicity [30] and found similar or better classification accuracy to the classic kernel based methods. Finally, as previously mentioned, Gilmer et al. [36] iterate upon existing GNN methods (reframed as message passing) to find the best configuration to predict molecular properties among the existing deep learning approaches.

#### DDI prediction

As introduced previously, polypharmacy is a promising treatment approach in the case of complex diseases, but with a cost: the possibility of undesirable interactions among co-administrated drugs, i.e. polypharmacy side effects. The appearance of side effects has often been reported by patients affected by multiple illnesses who have been treated with multiple drugs simultaneously. Since laboratory screenings of DDIs are very challenging and expensive, there is growing interest in studying and predicting drug interactions using computational methods. Therefore, this section will review some deep learning approaches that use biological networks to predict the interaction among drugs, which is usually formulated as a link prediction problem. As detailed below, the reviewed graph-based deep learning methods outperform, often in a significant way, the classic machine learning and deep learning methods used as baselines, showing that graph-based deep learning approaches can capture meaningful insights into the DDIs prediction problem.

Decagon [46] is an innovative GCN method for multi-relational link prediction which operates on large multimodal graphs where nodes, i.e. proteins and drugs, are connected through diverse kinds of edges according to the interaction type. These multimodal networks are constructed combining PPIs, DDIs and drug–protein interaction networks. Once the multimodal network is obtained, Decagon performs two main steps: an encoding and a decoding process. The first step is executed by a GCN, which takes in the graph and gives out a node embedding for it. The second step is carried out by a tensor factorization decoder, which obtains a polypharmacy side effects model from the embedding of the nodes given as input. One of the major strengths of Decagon is its capability of identifying not only the presence of an interaction between drugs, but also of which type. Decagon outperforms the state-of-the-art baselines, e.g. classic machine learning approaches for link-prediction, methods for representation learning on graphs and methodologies for multirelational tensor factorization, by an average of 20% and in some cases was as high as 69%. The authors, furthermore, note the importance of including the PPI network in such analysis. In fact, 68% of drug combinations have no common targets, suggesting that PPI information may represent a critical link to understanding which specific target drugs interact with proteins.

Another encoder-decoder method for multi-relational link prediction is presented in [27]. The proposed method, HLP, is designed to perform on a multi-graph representation of DDIs, defined as networks having drugs as nodes and multiple interactions as edges among node pairs. The characteristic which makes HLP an interesting method is its ability to capture the global graph structure in addition to the local neighborhood information. HLP shows enhanced performance when contrasted with similar multi-link prediction models and to Decagon [46]. However, Decagon is tailored to work on networks composed of relationships between drugs and also proteins, which according to Decagon’s authors is important to include, while HLP works and is tested on DDI networks only.

Ma et al. [43] propose yet another approach for DDI prediction by integrating multiple sources of information and using an attention mechanism to learn the appropriate weights associated with each view, resulting in interpretable drug similarity measures. They use a GCN architecture to build an autoencoder, with a GCN as the encoder and another GCN as the decoder. Each drug is a node in their graph, but it contains multiple graphs with the same nodes, and the edge in each view of the graph corresponds to the similarity between the node features in that view. Ultimately, they want to get a node embedding for each node in the graph and recover a single adjacency matrix that captures the information across views, which can predict drug to drug interactions. Ma et al.’s method is compared with several baselines, such as nearest neighbor, label propagation, multiple kernel learning and the non-probabilistic GAE model in [96]. Results show that [43] significantly outperforms the baselines for both the binary and multilabel prediction settings.

As previously mentioned, DDIs represent a promising research direction to find therapies for complex diseases. Therefore, besides the prediction of side effects from multiple drugs, many efforts are currently aimed at the discovery of polypharmacy treatments. Jiang et al. [55] propose an approach to predict synergistic drug combinations against different cancer cell lines. The authors formulate the problem as a link prediction task. The input is a heterogeneous network, diverse for each cancer cell line under study, obtained through the combination of synergistic DDI, DTI and PPI networks. The method, whose algorithm is based upon Decagon [46], presents a GCN encoder followed by a matrix decoder to predict the synergistic score among pairs of drugs. The method proposed by Jiang et al. [55] shows improved performance in comparison to an SVM, random forest, elastic net and feature-based deep learning methods. Additionally, it is comparable to a state-of-the-art approach very popular in the field. Finally, the authors apply the method to predict *de novo* combinations of drugs and discovered that some of them have been already reported in the literature as synergistic against cancer.

Another line of research leverages GCN methods for personalized drug combination predictions. An approach sharing this aim is GAMENet [44]. GAMENet combines the patient representation obtained by employing an embedding network followed by a dual recurrent neural network with the network information derived from a memory module. The latter is based on a GCN and captures information from two networks, namely the graph representation of longitudinal patient electronic health records (EHR) and a DDI network.

CompNet, proposed in [45], is another method for supporting doctors in the prescription of drug combinations. In particular, EHR data, prescribed drugs records and adverse DDI networks are used for learning patient and drug information representations which are then combined to obtain the prediction. The module encoding the drug information, referred to as a medicine knowledge graph representation module, is constructed using a relational GCN. Both GAMENet [44] and CompNet [45] are subjected to an ablation study to assess the importance of including DDIs information. In both cases, including the DDI network enhances the performance in a significant way. Furthermore, GAMENet and CompNet outperformed several state-of-the-art and classic machine learning approaches across various effectiveness measures, including F1, Jaccard coefficient and DDI rate. In addition, CompNet contrasts its performances to the ones achieved by GAMENet. CompNet outperforms GAMENet in terms of the Jaccard coefficient, recall, F1 and DDI rate, whereas GAMENet is superior only in terms of precision. CompNet’s authors claim that recall is more important than precision when the aim consists of recommending combinations of drugs. In reality, such prediction systems represent a support tool for doctors, and therefore the objective is to provide them with a wide and comprehensive screening of drugs co-administration possibilities, rather than with a precise but limited list.

A different way of handling DDI prediction is presented in [25]. The authors propose a method to enhance DDI extraction from texts by using a graph representation of the drugs under study. This approach concatenates the results of a CNN used on textual drug pairs with the ones obtained by applying a GCN on their graph molecular structure. Such an approach is motivated by the fact that a lot of information about interactions among different drugs is available in the literature but is not always reported in DDI databases or easily available when prescribing drugs, and at the same time the molecular structure encloses meaningful information for interaction prediction. Results show that [25] has comparable performance to deep learning state-of-the-art approaches, including Zeng et al. [113] on which [25] is based upon, which outperforms the classic machine learning methods used as baseline. Moreover, in [25] it is shown that including the information on the molecular structure enhances the text-based DDI predictions in a considerable way.

### Disease diagnosis

In the last few years, investigating disease diagnoses through deep learning has been of great interest to the research community. However, methods which use graphs, and in particular biological networks, are in a minority. The work proposed in [62] is situated in this small research area. The authors aim to predict lung cancer from a PPI network integrated with gene expression data by using a combination of spectral clustering and CNNs. The authors try different configurations of the proposed method to identify the one which performs the best and evaluate their method in terms of accuracy, precision and recall.

Additionally, Rhee et al. [72] propose another example of deep learning on biological networks to perform breast cancer sub-type classification. Their method integrates a GCN and a relational network (RN) and takes in a PPI network enriched with gene expression data. Exploiting the GCN, their approach is capable of learning local graph information, while the use of the RN permits capturing complex patterns among sets of nodes. The GCN and RN outputs are combined to obtain the classification results. The method is compared to SVMs, random forest, k-nearest neighbor and multinomial and Gaussian naive Bayes and performance is obtained through a Monte-Carlo cross validation experiment. The results show that the proposed method outperforms the baselines across all the used metrics, showing that learning PPI network feature-representation by means of a GCN may significantly help in capturing patterns in gene expression data.

Apart from performing disease diagnosis using the biological networks described in the introduction, there are also studies that use different types of networks, such as RNA-disease associations or graphs obtained by converting biomedical images, in combination with deep learning techniques. Deep learning is gaining traction nowadays in the disease diagnosis research area and so we report on some of these approaches in the following paragraphs to demonstrate how broad this field is, despite using networks that are not conventionally considered biological networks.

The next two examples are applications which employ RNA-disease and gene-disease association networks respectively. Zhang et al. [114] propose a method whose input is a graph representing the association among diseases and RNAs, named a RNAs-disease network. The authors use a GCN combined with a graph attention network to capture both the global and the local structure information of the input, with the objective of predicting RNA-disease associations. Instead, the objective of Han et al. [115] is to predict gene-disease associations. To this aim, the authors propose a combination of two GCNs and a matrix factorization. Diseases, gene features and similarity graphs are given to two parallel GCNs, which combine their obtained embeddings through an inner product to obtain the prediction. Both [114] and [115] show their effectiveness in capturing useful information from the RNAs- or gene-disease association networks in respect to the methods used as baselines.

Besides that, research in this field has centered around converting biomedical images to a graph and then performing classification. For example, Zhang et al. [116] predict Parkinson’s Disease from a graph representation of multimodal neuroimages using a classifier based on a GCN. Marzullo et al. [117] present a GCN working on a graph mapping of MRI images to predict Multiple Sclerosis. The use of GCNs enhances the performance with respect to the machine learning and/or deep learning baselines for both [116] and [117], showing the potential improvements that GCNs can yield in the image analysis research area.

Another example is [118], whose aim is breast cancer diagnosis from mammogram images, with only a few labeled samples. They are able to create pseudo-labels for the unlabeled images via graph-based semi-supervised learning, where each node is an image and the edge represents the similarity between images. A CNN is then trained on the individual images using the true and pseudo-labels. This method introduces a valuable contribution in the area of medical image analysis with deep learning, where large datasets are required for the training to be effective. Specifically, the authors develop a strategy to overcome a typical limitation in the field: having few labeled data points. They use instead an algorithm which allows for the inclusion of unlabeled data in the training procedure of the deep learning model. Results show the merits of this strategy, which drastically enhances the performance.

### Metabolic networks and GRNs

While less extensively studied, GNNs have also been used for analyzing metabolic and GRNs. These early studies have reported promising results, showing that deep learning’s capability to capture non-linearity in the data can positively affect the study of these complex and meaningful biological networks.

**Metabolic Networks** Studying and reconstructing metabolic pathways is a key aspect of obtaining a better understanding of physiological processes, drug metabolism and toxicity mechanisms and others. To the best of our knowledge, the literature lacks papers investigating this network using graph-based deep learning methods. It is possible to find plenty of work aiming to analyze, model and reconstruct metabolic pathways, or whose objective is to predict drug metabolism, but they use classical tools [119]. Two recent papers, namely [52] and [51], fit our review topic. The method in [52] aims to predict the metabolic pathway to which a given compound belongs by means of a hybrid approach. It uses a GCN to learn the shape feature representation of a given molecular graph, which then is the input to a random forest to perform classification. The authors compare their method with several state-of-the-art machine learning approaches, showing the positive impact of employing GCNs as a means for capturing insights from the graph representation of the molecules under study. Furthermore, the authors develop a methodology to interpret the feature representation provided by the GCN in terms of chemical structure parameters, such as the diameter.

The objective of the work presented in [51] is different. The authors aim at predicting the dynamical properties of metabolic pathways by leveraging their graph representation’s structure using a GNN framework. The graph representing the pathway is a bipartite graph obtained from systems biology markup language models of biochemical pathways using a Petri net modeling approach [120]. The authors contrast the proposed method with a classifier predicting the majority class in the test set and report that their method always outperforms the baseline. The method in [51] represents a computationally efficient alternative to the onerous numerical and stochastic simulations which are often used for assessing the dynamical properties of biochemical pathways.

**Gene Regulatory Networks** Knowledge about GRNs is essential to gain insights about complex cellular mechanisms and may be useful for the identification of disease pathways or new therapeutic targets. Therefore, GRNs are widely investigated, with particular interest bestowed upon inferring, validating and reconstructing them. Such investigations are mostly performed with classic methods, while the amount of developed graph-based deep learning approaches is rather small, as for metabolic networks. To date, curated GRN datasets are not yet available or are difficult to obtain for a large number of organisms [49, 121]. For this reason, GRNs are mostly analyzed with unsupervised methods [121], since supervised techniques, and deep learning in particular, require a large number of well annotated samples in order to be effective. Additionally, GRN inference is usually accomplished by employing information from gene expression data, which are intrinsically noisy [122] and therefore not ideal for training models. However, some deep learning models, specifically RNNs, report promising results, although they do not use any kind of graph information to perform the task. One example is the work in [122], which enhances the training quality by introducing a non-linear Kalman filter, which deals very effectively with the noise in the data.

Despite the limitations discussed above, Turki et al. [49] present an example of graph-based deep learning approaches. The authors use an unsupervised method to obtain a preliminary version of the GRN from gene expression time series data, which is denoised through a cleaning algorithm, and then used to train diverse supervised methods to perform link prediction among gene pairs. The proposed data cleaning algorithm is of crucial importance and could positively impact the field of GRN analyses since it increases the quality of the GRN data. More in detail, the denoised features are obtained by projecting the original features onto the eigenvectors of the distance matrix of the feature vectors calculated using the Laplacian kernel function. The supervised methods Turki et al. use after cleaning the GRN includes SVMs and deep learning approaches, such as a DNN and a deep belief network. The latter two outperform the unsupervised state-of-the-art baseline, although failed to outperform the linear SVM-based approach.

## Discussion

The promise of deep learning, based on its success in other fields [7, 8], is now also being seen across many different areas of biological network analysis. The methods we reviewed reported to consistently match or beat previous state-of-the-art methods using classical machine learning algorithms, providing evidence of one of deep learning’s core advantages: its strong empirical classification performance.

Another advantage of deep learning is its ability to effectively deal with large datasets [123], which can be challenging for classical machine learning methods [123, 124]. Although the training process of deep learning models with huge amounts of data is a non-trivial task, the advances in parallel and distributed computing have made training these large deep learning models possible [125, 126]. The large number of matrix multiplications, high memory requirements and easy parallelizability of neural networks have been particularly well served by the recent breakthroughs in GPU computing [2, p. 440].

Finally, given that deep learning is a learning approach based on a hierarchy of non-linear functions, it is capable of detecting patterns in the raw data without explicit feature engineering. While it is not the only method that can handle non-linear relationships, the composition of many simple, non-linear layers makes it particularly adept at learning patterns at different layers of abstraction [126], enabling more complex patterns to be detected.

While deep learning methods are very promising, there are limitations and many open questions to be solved. One of the main problems with deep learning is its lack of interpretability. While there has been some recent progress in this area [127, 128], the black box nature of deep learning algorithms remains a key challenge, particularly in bioinformatics, where one is interested in understanding the mechanisms underlying the biological processes [129, 130]. Additionally, interpretability is critical in the context of models that guide medical decisions, where doctors and patients are often unlikely to trust the output of a deep learning model without sufficient understanding of the prediction process [127].

Another issue is the need for large labeled datasets, since deep neural networks have a large amount of hyperparameters to tune. Although the recent advances in the technology enable the collection of huge amounts of data, the field of bioinformatics often suffers from quality issues with the data and the lack of reliable labels, since much of the data is unlabeled [127]. In such a scenario, training can be difficult and can limit the effectiveness of deep learning in bioinformatics, which can be seen for example in GRN analysis. Furthermore, not all application areas in bioinformatics have access to large amounts of data. In disease diagnosis, for example, data points can represent individual patients and therefore amassing the large datasets necessary for deep learning to excel can be challenging. Furthermore, the access to disease-related data is often limited by privacy restrictions [131], therefore contributing to the limited size of datasets in the field [132]. In such smaller data regimes, classical machine learning methods, which are often available in standard programming libraries, can be a suitable alternative [133], such as graph kernels [98, 102, 134, 135] and their implementations [136].

Despite these challenges, deep learning on graphs is an active area of research and is already achieving exciting results across various bioinformatics disciplines such as proteomics, drug development and discovery, disease diagnosis and more, as we have seen in this review. We can therefore anticipate the continued development of new algorithms, both within and outside bioinformatics, that can be used to analyze biological networks. Moreover, the amount of data generated from recent advancements in high-throughput technology will continue to grow, providing even more opportunities for deep learning to solve existing as well as new problems in biological network analysis.

**Key Points**

- Biological networks are a meaningful way of representing many biological processes, such as PPI networks, DDI networks and GRNs, because they can model both the biological entities as well as the relationships between those entities.
- The graph representation of biological networks enables the formulation of classic machine learning tasks in bioinformatics, such as node classification, link prediction and graph classification.
- Deep learning methods on graphs, specifically GNNs, are a new way of solving these tasks by capturing hierarchical non-linearities in the data and neighborhood information represented by the network.
- GNNs have been successfully applied in several areas of bioinformatics such as protein function prediction in proteomics and polypharmacy prediction in drug discovery & development.
- GNNs are also being used to tackle questions across various emerging applications of bioinformatics, such as metabolic pathway prediction in metabolic network analysis.

## Supplementary Material

main_Table_2_as_Supplementary_file_bbaa257

## Notes

**Giulia Muzio** is a PhD student at the Machine Learning and Computational Biology Lab at ETH Zürich, whose main research interest is computational biology.

**Leslie O’Bray** is a PhD student at the Machine Learning and Computational Biology Lab at ETH Zürich, whose main research focus is machine learning on graphs.

**Karsten Borgwardt** is a Full Professor of Data Mining in the Life Sciences at ETH Zürich since 2017 and the recipient of the 2013 Alfried Krupp Award.
