# Supplementary material for: Biological network analysis with deep learning
Source: Brief Bioinform. 2020 Nov 10;22(2):1515–30. doi: 10.1093/bib/bbaa257 (PMC7986589; doi:10.1093/bib/bbaa257)
Supplement: main_Table_2_as_Supplementary_file_bbaa257 [file main_table_2_as_supplementary_file_bbaa257.pdf]

Table 2. This table reports for each of the reviewed method the website where to find the implementation, if available.

	Method	Implementation
GNNs	Bruna et al. [93]	–
	Defferrard et al. [94]	https://github.com/mdeff/cnn_graph
	Duvenaud et al. [95]	http://github.com/HIPS/neural-fingerprint
	Gilmer et al. [36]	–
	Grover & Leskovec [54]	https://github.com/aditya-grover/node2vec
	Hamilton et al. [64]	https://github.com/williamleif/GraphSAGE
	Kipf & Welling [96]	https://github.com/tkipf/gcn
	Niepert et al. [30]	–
	Perozzi et al. [89]	https://github.com/phanein/deepwalk
	Tang et al. [90]	https://github.com/tangjianpku/LINE
Proteomics	Gligorijević et al. [71]	https://github.com/VGligorijevic/deepNF
	Grover & Leskovec [54]	https://github.com/aditya-grover/node2vec
	Hamilton et al. [64]	https://github.com/williamleif/GraphSAGE
	Liu et al. [60]	–
	Senior et al. [76]	https://github.com/deepmind/deepmind-research/tree/master/alphafold_casp13
	Yue et al. [41]	https://github.com/xiangyue9607/BioNEV
	Zeng et al. [56]	https://github.com/CSUBioGroup/DeepEP
	Zhang & Kabuka [100]	–
	Zitnik & Leskovec [65]	https://github.com/marinkaz/ohmnet
Drug Development, Discovery and Polypharmacy	Asada et al. [25]	–
	Duvenaud et al. [95]	http://github.com/HIPS/neural-fingerprint
	Feinberg et al. [108]	–
	Fout et al. [30]	https://github.com/fouticus/pipgcn
	Gilmer et al. [36]	–
	Jiang et al. [55]	–
	Kearnes et al. [33]	–
	Li et al. [112]	https://github.com/MingCPU/DeepChemStable
	Liu et al. [111]	–
	Ma et al. [43]	–
	Manoochehri et al. [40]	–
	Niepert et al. [30]	–
	Shang et al. [44]	https://github.com/sjy1203/GAMENet
	Stokes et al. [23]	https://github.com/swansonk14/chemprop
	Torng & Altman [26]	–
	Vaida & Purcell [27]	–
	Wang et al. [45]	https://github.com/WOW5678/CompNet
	Zeng et al. [28]	https://github.com/ChengF-Lab/deepDTnet
	Zitnik et al. [46]	http://snap.stanford.edu/decagon
Disease Diagnosis	Han et al. [115]	–
	Marzullo et al. [117]	–
	Matsubara et al. [62]	https://sites.google.com/site/nacherlab/analysis
	Rhee et al. [72]	–
	Sun et al. [118]	–
	Zhang et al. [114]	–
	Zhang et al. [116]	https://github.com/sheryl-ai/MVGCN
MN & GRN	Baranwal et al. [52]	https://github.com/baranwa2/MetabolicPathwayPrediction
	Bove et al. [51]	–
	Turki et al. [49]	–
